# Antibacterial Properties of Tough and Strong Electrospun PMMA/PEO Fiber Mats Filled with Lanasol—A Naturally Occurring Brominated Substance

**DOI:** 10.3390/ijms150915912

**Published:** 2014-09-09

**Authors:** Richard L. Andersson, Antonio Martínez-Abad, José M. Lagaron, Ulf W. Gedde, Peter E. Mallon, Richard T. Olsson, Mikael S. Hedenqvist

**Affiliations:** 1 KTH Royal Institute of Technology, School of Chemical Engineering and Science, Fibre and Polymer Technology, 100 44 Stockholm, Sweden; E-Mails: riander@kth.se (R.L.A.); gedde@kth.se (U.W.G.); rols@kth.se (R.T.O.); 2 Novel Materials and Nanotechnology Group, Institute of Agrochemistry and Food technology (IATA), Spanish Council for Scientific Research (CSIC), Avda. Agustín Escardino 7, 46980 Burjassot, Spain; E-Mails: antma@kth.se (A.M.-A.); lagaron@iata.csic.es (J.M.L.); 3 Department of Chemistry and Polymer Science, University of Stellenbosch, Private Bag X1, Matieland 7602, South Africa; E-Mail: pemallon@sun.ac.za

**Keywords:** electrospinning, antimicrobial/antibacterial, Lanasol, nanofiber toughness, sea algae, poly (methyl methacrylate) (PMMA)/polyethylene oxide (PEO)

## Abstract

A new type of antimicrobial, biocompatible and toughness enhanced ultra-thin fiber mats for biomedical applications is presented. The tough and porous fiber mats were obtained by electrospinning solution-blended poly (methyl methacrylate) (PMMA) and polyethylene oxide (PEO), filled with up to 25 wt % of Lanasol—a naturally occurring brominated cyclic compound that can be extracted from red sea algae. Antibacterial effectiveness was tested following the industrial Standard JIS L 1902 and under agitated medium (ASTM E2149). Even at the lowest concentrations of Lanasol, 4 wt %, a significant bactericidal effect was seen with a 4-log (99.99%) reduction in bacterial viability against *S. aureus*, which is one of the leading causes of hospital-acquired (nosocomial) infections in the world. The mechanical fiber toughness was insignificantly altered up to the maximum Lanasol concentration tested, and was for all fiber mats orders of magnitudes higher than electrospun fibers based on solely PMMA. This antimicrobial fiber system, relying on a dissolved antimicrobial agent (demonstrated by X-ray diffraction and Infrared (IR)-spectroscopy) rather than a dispersed and “mixed-in” solid antibacterial particle phase, presents a new concept which opens the door to tougher, stronger and more ductile antimicrobial fibers.

## 1. Introduction

Electrospinning is a simple technique to continuously generate ultra-fine high surface area fibrous mats with fiber diameters ranging from tenths of nanometers to several microns. Their porous nature and the suitability of the technique for incorporating other materials within the fibers at the nanoscale level have prompted their use in a wide range of applications, such as: wound dressings, implant materials, or tissue scaffolds with high biocompatibility and antimicrobial capacity [1]. In this context, previous studies on antimicrobial electrospun fibers have extensively focused on the inclusion of silver nanoparticles (AgNPs) inside the fiber mats [2,3,4,5,6,7], since silver has a higher microbial toxicity than mercury, copper and cadmium [8]. AgNPs have also been shown to be one of the most effective antimicrobial agents for bacteria, viruses, and other microbial organisms [9]. Examples of polymers used for this purpose include polyethylene oxide (PEO), polyacrylonitrile (PAN), nylon-6, polyurethane (PU), polycaprolactone (PCL), poly(lactic-*co*-glycolic acid) and poly(lactic acid) (PLA) [2,3,4,5,6,10]. However, a drawback with electrospinning fibers containing conductive AgNPs is that the fiber morphologies and average fiber diameter depend on the solid particle concentration, which frequently result in the formation of non-uniform fibers that are fragile due to beading, splaying and improper embedding of the particle phase [3,4,6]. Even more critical is the recent concerns regarding the toxicity of AgNPs. The particles have been shown to exert detrimental effects in humans and other mammals, including dysfunction of mitochondria and damage of DNA [11,12]. The following was stated by Windler *et al.* [13]; “*The development of new and better antimicrobials is an ongoing topic of research and antimicrobials based on naturally-derived products are increasingly discussed*”. Hence, alternative strategies to obtain antimicrobial fibrous mats that are not relying on inclusion of an embedded nanoparticle phase are, therefore, highly motivated.

**Figure 1:**
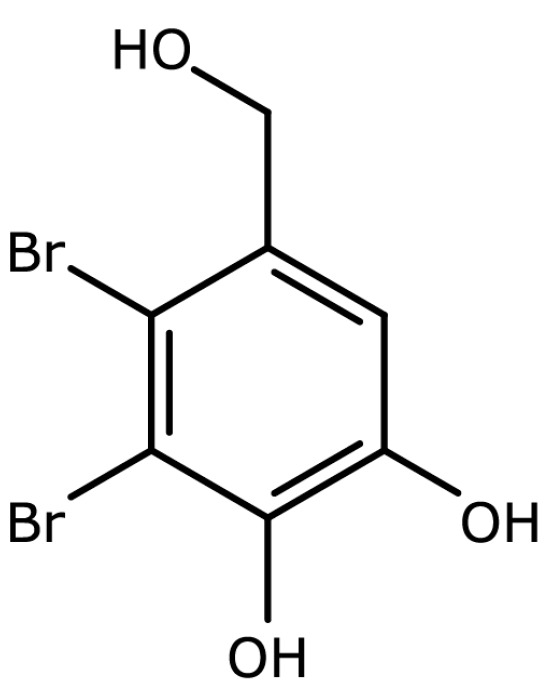
Lanasol structure, (2,3-dibromo-4,5-dihydroxybenzyl alcohol).

The antimicrobial substance Lanasol (2,3-dibromo-4,5-dihydroxybenzyl alcohol, see Figure 1) exists in the red alga *Rhodomela confervoides* [14], as well as in several other species in the *Rhodomelaceae* family [15], and also in *Polysiphonia lanosa* [16]. Although its antibacterial effect against both gram-negative and gram-positive bacteria has been documented [15], to the best of our knowledge, no studies have been reported on the use of this substance as an additive in any kind of polymer.

In this article, we present the first fiber system incorporating the naturally occurring antimicrobial agent Lanasol, which was prepared by electrospinning. It is demonstrated that the antibacterial properties against *S. aureus* are very significant, and only small amounts of dissolved Lanasol (4 wt %) are needed for a 4-log reduction (99.99%) in bacterial viability. A blend of poly (methyl methacrylate) (PMMA) and polyethylene oxide (PEO) was selected as the polymer matrix due to its good biocompatibility [17,18,19], miscibility [20,21,22], and toughness [23]. At higher contents of Lanasol (above 4 wt %), even greater antimicrobial efficacy was observed, at the same time as the toughness of the fiber mats was unaffected, which is in contrast to all fiber systems relying on embedded solid antimicrobial fillers.

## 2. Results and Discussion

### 2.1. Fiber Morphology

**Figure 2:**
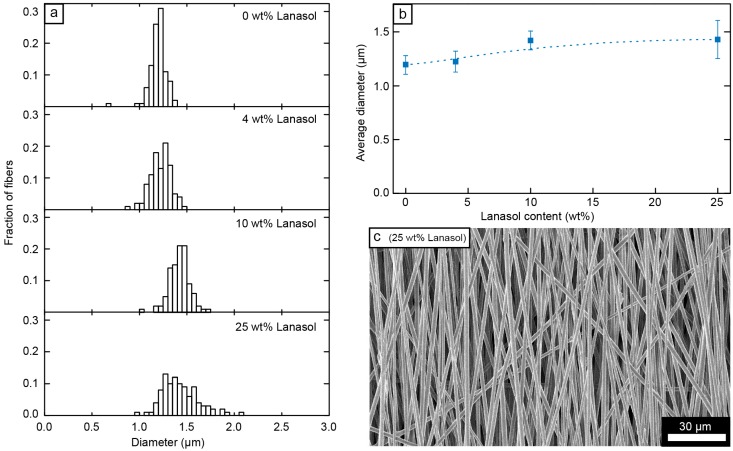
(**a**) Fiber diameter distributions for the 75/25 PMMA/PEO fibers with different amounts of Lanasol; (**b**) average fiber diameter (error bars indicate one standard deviation) as a function of Lanasol content; and (**c**) scanning electron micrograph of the fibers with 25 wt % Lanasol.

Fibers consisting of 75 wt % PMMA and 25 wt % PEO with different amounts of added Lanasol (0–25 wt %) were electrospun from DMF as solvent onto a rotating collector to align the fibers (Figure 2c) (the average deviation from perfect alignment was 14° ± 1° for all the fiber systems). The distributions of the diameters of these fibers are given in Figure 2a and their averages are displayed in Figure 2b, showing a small increase in fiber diameter (1.2 ± 0.09 to 1.4 ± 0.19 µm) as increasing amounts of Lanasol were added.

**Figure 3:**
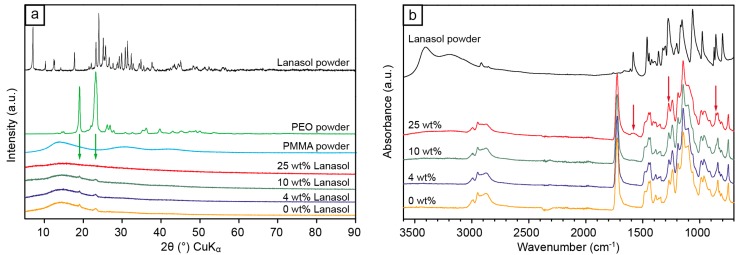
(**a**) Wide angle X-ray diffractograms of the PMMA/PEO electrospun fibers with different amounts of Lanasol, compared to pristine powders of Lanasol, PEO and PMMA; and (**b**) Infrared (IR)-spectrograms of the same fiber mats compared to the pristine Lanasol. Arrows at 1585, 1280 and 860 cm^−1^ indicate the appearance of new peaks from the Lanasol when dissolved into the fibers.

The pristine Lanasol was found to be crystalline (Figure 3a), with a broad range of different lattice planes and plane spacings that lead to X-ray diffraction (Bragg’s law gives distances from 1.64 to 12.6 Å). The PMMA/PEO blend without Lanasol was essentially amorphous, except for small peaks at 4.63 and 3.79 Å (2θ: 19.2° and 23.5°), which originated from the 2.1% PEO crystallinity, as determined from the ratio of the areas of these deconvoluted peaks to the total area of the sample [22]. As the Lanasol content increased, the crystalline fraction gradually decreased until no crystallinity could be detected (25 wt %). This indicates that the presence of the antimicrobial compound hindered the crystallization of PEO, resulting in an increased miscibility of the PEO and the PMMA with an evenly distributed and dissolved Lanasol component.

Infrared (IR) spectroscopy of the fiber mats (Figure 3b) with different amounts of Lanasol showed a clearly increased IR-absorption ascribed to OH-groups (3400–3100 and 1280 cm^−1^), C=C–C (aromatic ring stretch, 1585 cm^−1^), C–H (aromatic substituted, 860 cm^−1^), with increasing Lanasol content [24,25]. The bands associated with the bonds in the polymer matrices remained unchanged, indicating that no degradation of the polymers had occurred during processing in the presence of Lanasol.

### 2.2. Antimicrobial Tests

**Figure 4:**
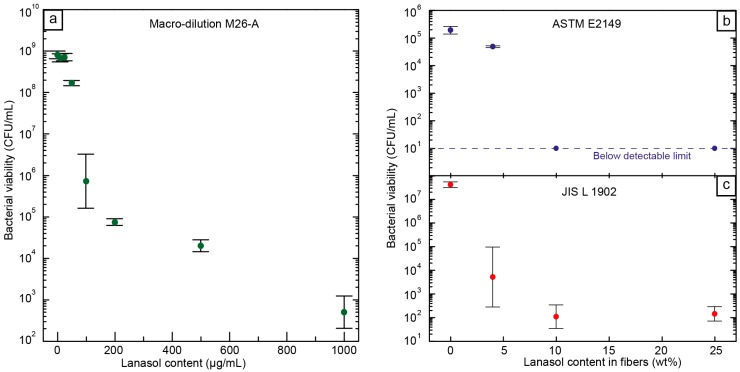
Bacterial viability, (**a**) after macro-dilution with Lanasol in solution; (**b**) in electrospun fibers containing different amounts of Lanasol suspended in solution (ASTME2149) [30]; and (**c**) after bacterial contact measurements on the surface of the fiber mat (JISL1902) [31].

Although an antimicrobial capacity of Lanasol [15,26] and its native plant family *Rhodomelaceae* [27] has previously been reported, breakpoints for the tested microorganism have not yet been established. A macro-dilution technique was therefore employed to evaluate its efficacy against *S.* *aureus* and to determine the minimum inhibitory concentration (MIC) and minimum bactericidal concentration (MBC) (Figure 4a). Even in a nutrient-rich environment, such as Tryptic soy broth (TSB), a concentration of 0.01 mg/mL, *i.e.*, 10 ppm (MIC), Lanasol was sufficient to inhibit bacterial proliferation for at least 20 h. Below that MIC, the culture was able to grow to values similar to those of the controls without the antimicrobial agent. Above a concentration of 0.1 mg/mL, *i.e.*, 100 ppm (MBC), a reduction of >99.9% in the viability of the bacterial population was observed. This antimicrobial effectiveness is relatively high as compared to other natural antimicrobial compounds, and similar to that of silver under the stated conditions [28,29].

The antimicrobial performance of the PMMA/PEO fibers containing Lanasol was evaluated both after immersing the fibers in an aqueous bacterial suspension under agitation (ASTM E2149, Figure 4b) and after adsorption onto the fibers’ surfaces (JIS L 1902, Figure 4c) [30,31]. As the fiber mats are highly porous, their surface effectiveness had to be tested in accordance with the textile standards, by absorbing a bacterial suspension onto the fibers. Fiber mats without Lanasol allowed a proliferation of the bacterial culture within the limitations of the diluted growth medium and the viability values were not significantly different from those of the controls without fibers. The fibers containing Lanasol, on the other hand, showed good antibacterial properties in all cases. According to JIS L 1902 [31], fibers containing 4 wt % Lanasol produced a viability reduction of *ca.* 4 orders of magnitude (99.99% reduction), *i.e.*, a strong bactericidal effect [32]. All the fiber samples with 10 wt % Lanasol or higher resulted in a decrease of more than five orders of magnitude in bacterial viability. Under the dynamic contact measurement (ASTM E2149) [30], viable counts of bacterial colonies were below the detectable threshold for fiber mats with ≥10 wt % Lanasol. However, for samples with only 4 wt % Lanasol, a smaller but significant reduction of 75% of the bacterial population was achieved. These results showed the high antimicrobial activity of the tested fibers against a common pathogen, such as *S. aureus*, and emphasize the potential of these fibers for use as, e.g., textiles and membranes, especially in hospital environments where *S. aureus* is the leading cause of hospital-acquired infections (HAI) throughout the world [33].

### 2.3. Mechanical Properties

**Figure 5:**
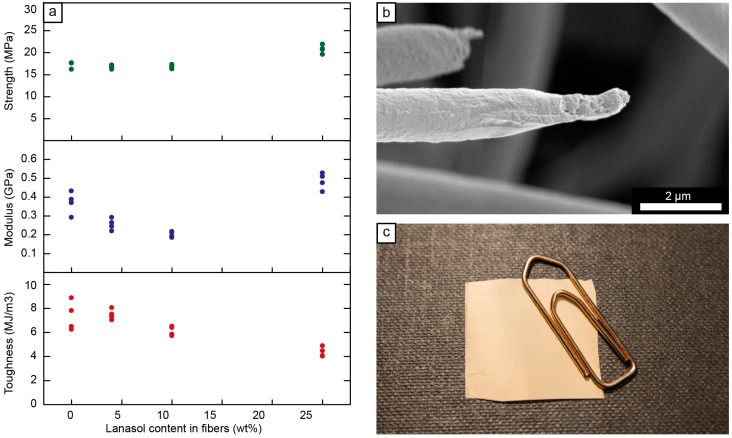
(**a**) Maximum stress, modulus and toughness as a function of Lanasol content in the electrospun PMMA/PEO fiber mats; (**b**) Scanning electron micrograph of a fiber fracture surface post tensile test (25 wt % Lanasol); and (**c**) Photograph of a smooth electrospun fiber mat (2 × 2 cm^2^) consisting of *ca.* 200,000 aligned fibers, note the slight orange tint due to the Lanasol (10 wt %).

The fiber mat with 4 wt % Lanasol showed equivalent mechanical properties with the neat PMMA/PEO (75/25 wt %) fiber mat and the highest toughness among the tested formulations (7.5 MJ/m^3^). The toughness decreased only marginally when more Lanasol was added to the polymer (Figure 5a), but despite this decrease in toughness, the fiber mats still showed a toughness more than one order of magnitude higher than that of electrospun fibers consisting of only PMMA [34]. The cause for the small decrease in toughness when as much as 25 wt % Lanasol was added is evident in Figure 5b, which shows inhomogeneities at the fracture surfaces of the fibers. A continuous necking with propagation along the fibers could therefore not occur during the fiber deformation, which was the case for the fibers with lower contents of Lanasol [23]. However, even in the absence of a necking phenomenon, Figure 5a shows that the modulus and the maximum stress exhibited were the highest for the formulation with 25 wt % Lanasol, indicating a stiffening/strengthening effect for this composition. This sample showed no signs of crystallinity (Figure 3a), in contrast to the small PEO crystallinity observed in the other samples, which suggests that the higher modulus and strength was related to a greater degree of molecular alignment in this sample [35]. All samples showed a strain to failure of more than 30%, and samples with 0–10 wt % Lanasol showed a strain to failure of more than 50%. These values are considerably higher than those for fibers based on the same matrix but with solid fillers, where crack initiation and fiber failure occurred at the particle-fiber interface [23].

In summary, the Lanasol in the fibers had little effect on the mechanical properties due to its compatibility and solubility in the polymers, which was also indicated by the smooth and uniform fiber mat morphology shown in Figure 5c. These desirable mechanical properties stem, not only from the single-phase nature of the fibers, but also from the absence of any sedimentation/agglomeration or inhomogeneity of the solutions prior to the electrospinning.

## 3. Experimental Section

### 3.1. Preparation of Fiber Solutions

Powders of PMMA with a *M*_w_ of 410 kDa (Alfa Aesar, Ward Hill, MA, USA) and PEO (Acros Organics, Geel, Belgium) with a *M*_w_ of 600 kDa were mixed together in the proportions of 75 wt % PMMA and 25 wt % PEO. The mixtures were then dissolved in dimethylformamide (DMF, BDH Prolabo 99.8%, Istanbul, Turkey); 90 wt % DMF with respect to the total polymer content), and various amounts (0–25 wt %) of Lanasol (2,3-dibromo-4,5-dihydroxybenzyl alcohol, AApin Chemicals Limited, Oxon, UK) with respect to the total polymer mass, were then dissolved into this mixture (Figure 1). These solutions were heated to 70 °C and kept at this temperature under constant agitation for 2 h until homogeneous and transparent solutions were obtained. They were then electrospun within 24 h at 22 °C and *ca.* 25% relative humidity.

### 3.2. Electrospinning and Collection of Fiber Mats

The electrospinning solutions were electrospun from a 5 mL solvent-resistant polypropylene syringe at a rate of 10 µL/min (±0.1%), via a PTFE tube, through a flat tip 18-gauge stainless steel needle with an internal diameter of 0.84 mm. The needle tip was positioned 25 cm vertically above a rotating collector (50 mm diameter), and the electrical potential from the needle to the collecting surface was maintained at *ca.* 11 kV. The cylindrical collector was spun at a rate of 2000 rpm in order to align the fibers during spinning. Infrared spectroscopy was performed on all samples prior to further characterization, in order to ensure that no solvent remained inside the fibers.

### 3.3. Bacterial Strain and Growth Conditions

*Staphylococcus aureus* subsp. *aureus* CECT 239 (ATCC 6538) was obtained from the Spanish Type Culture Collection (CECT; Valencia, Spain) and stored in Phosphate Buffer Saline (PBS) with 10% Tryptone Soy Broth (TSB, Conda Laboratories, Madrid, Spain) and 10% glycerol at −80 °C until needed. This species was chosen as model pathogen in this study since it is a type strain for *S. aureus*, and the recommended microorganism in the Japanese Industrial Standard L 1902 “*Antimicrobial Fabric Test*” (JIS L 1902 [31]) and in the equivalent ISO 20743 “*Determination of antibacterial activity of textile products*” [36] and AATCC 100“*Assessment of Antibacterial Finishes on Textiles*” [37] standards.

For experimental use, the stock culture was maintained by regular subculture to Tryptone Soy Agar (TSA; Conda Laboratories, Spain) at 4 °C and transferred monthly. Prior to each study, a loopful of bacteria was transferred to 10 mL of Tryptic soy broth (TSB; Conda laboratories, Madrid, Spain) and incubated at 37 °C overnight. A 100 μL aliquot from the overnight culture was then transferred to TSB and grown at 37 °C to the mid-exponential phase of growth (optical density absorbance value of 0.20 at 600 nm). This culture was diluted in the appropriate medium to achieve the initial inoculum concentration.

### 3.4. Antimicrobial Tests

Three different tests were carried out to test the antimicrobial performance of the Lanasol both as a powder and on electrospun fiber mats with various amounts of Lanasol (0–25 wt %). First, the minimal inhibitory concentration (MIC) and minimal bactericidal concentration (MBC) of the antimicrobial compound Lanasol were evaluated following the broth macro-dilution method M26-A described by the Clinical and Laboratory Standards Institute (CLSI). Briefly, a bacterial suspension in a mid-exponential phase was inoculated into 10 mL of TSB starting with an initial inoculum size of approximately 5 × 10^5^ CFU/mL. Different concentrations of the Lanasol powder were added and the tubes were incubated at 37 °C for 20 h. Then, 0.1 mL of each sample was sub-cultivated on TSA plates for viable count.

Secondly, the ASTM E2149 “Standard Test Method for Determining the Antimicrobial Activity of Antimicrobial Agents Under Dynamic Contact Conditions” [30], and the JIS L 1902 “Antimicrobial Fabric Test” [31] were followed to determine the antimicrobial performance of the Lanasol-containing fibers both under dynamic contact and on the surface of the mats. For the ASTM E2149 [30], 58 cm^2^ of the fibers were immersed in 50 mL of diluted nutrient broth with a bacterial inoculum of 3 × 10^5^ CFU/mL. The flasks were incubated for 18–20 h at 37 °C under agitation and the number of viables was determined by a traditional plate count as before. In JIS L1902 [31], the bacterial suspension was absorbed onto the surface of the porous fiber mats (5 × 5 cm^2^), incubated at 37 °C for 18–20 h and re-suspended in PBS, the number of viables was then determined by plate count. All experiments were performed in triplicate and the statistical significance of differences in bacterial viability values was determined in all cases on the ranks with a one-way analysis of variance (ANOVA) and Tukey’s multiple comparison tests. In all cases, a value of *p* < 0.05 was considered to be significant.

### 3.5. Tensile Testing of Fibers

**Figure 6:**
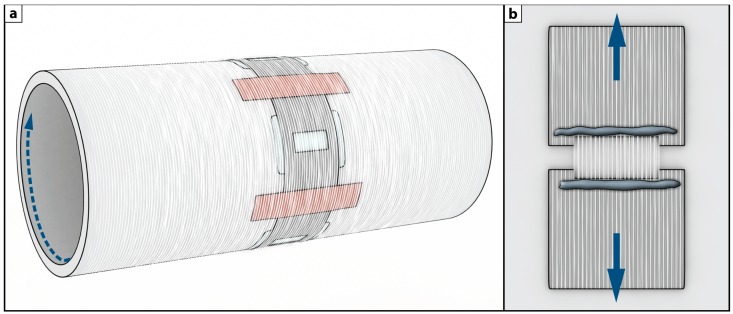
(**a**) Template transfer configuration, illustrating the aligned fibers on the rotating collector and the aluminium foil cutout template; and (**b**) Illustration of the fibers fixed on the template as mounted in tensile stage during tensile testing. Reprinted from Nature Publishing Group, with permission from Andersson *et al.* [23].

The tensile measurements were performed on a modified Deben Microtest stage, according to a previously published method [34]. In short, a strain rate of 0.5 mm/min (10% of sample length per min.) was used, and the tensile stress was calculated by dividing the measured force by the cross sectional area of the fiber mat. The cross sectional area was derived from the mass of a known area of the fiber mat (taken adjacent, in the rotating direction, to the tested fibers on the collector), and the density of the fibers (1.18 g/cm^3^). The fiber mats were collected on the rotating collector that had been prepared with a layer of fluorinated ethylene propylene release film (thickness = 76 µm), followed by a precut aluminum foil template (thickness = 30 µm) over the release film. After the spinning the electrospun fibers was glued to this aluminum template foil, and then moved to the tensile stage without being touched by hand, to avoid the risk of damage to the delicate thin mats. Only fiber mats that broke in the middle, *i.e.*, not adjacent to the glue border, were considered. Figure 6 illustrates this method and shows how the two sides of the template were cut after being mounted in the tensile clamps [34].

### 3.6. Electron Microscopy

A low voltage high resolution Hitachi S-4800 cold–field–emission scanning electron microscopy (FE-SEM) (Tokyo, Japan) was used, in order not to damage the thin fibers during observation (1 kV accelerating voltage). The fiber mats were coated with a *ca.* 2 nm layer of a conductive platinum–palladium (60/40) alloy, using a Cressington 208HR high-resolution sputter (Watford, UK) at 80 mA ion current for 20 s.

### 3.7. X-ray Diffraction (XRD)

XRD measurements were conducted on a PANalytical X’Pert Pro diffractometer (Almelo, The Netherlands) using Cu–Kα radiation (45 kV, 35 mA) and a 1.00 arcmin step size. The crystallinity of the samples was calculated from the ratio of the areas of the deconvoluted crystalline peaks to the total area of the diffractograms.

### 3.8. Infrared (IR) Spectroscopy

IR spectroscopy was performed on a Perkin-Elmer Spectrum 2000 (Waltham, MA, USA) using a 1 cm^−1^ scan step with a single reflection attenuated total reflectance stage (ATR) MKII Golden Gate unit (Specac Ltd., London, UK).

## 4. Conclusions

Naturally occurring and scarcely studied Lanasol (a cyclic brominated substance from red sea algae) was investigated as an antibacterial agent and breakpoints for its efficiency (MIC and MBC) was established against the most common cause for hospital-acquired (nosocomial) infections (HAI), *i.e.*, *S.* *aureus*. The Lanasol was found to be completely soluble in the PMMA/PEO polymer matrix (X-ray diffraction, IR-spectroscopy), which allowed electrospinning of uniform antimicrobial fibers of similar diameters for a range of Lanasol concentrations. According to JIS L 1902 [31] for antimicrobial contact measurements, the PMMA/PEO fibers with 4 wt % Lanasol resulted in a 4-log reduction (99.99%) in bacterial viability compared to the reference fibers without Lanasol. Antimicrobial activity under dynamic contact conditions (constant agitation in suspension, ASTM E2149 [30]), showed that fibers with ≥10 wt % Lanasol resulted in a total removal of all measurable quantities of bacteria. The mechanical properties of the fibers were almost unaffected even for high concentrations of Lanasol, up to 10 wt %. The toughness, in particular, was more than one order of magnitude higher than that for neat PMMA. A strain to failure of more than 50% was measured for these fiber mats, which is an essential property for fabrics that are intended as wound dressings, as they allow for application and transfer of patches that can follow the natural movement of the patient without breakage. The presented concept of using naturally occurring soluble antimicrobial brominated compounds may present a change in the 20-year-old paradigm that regards solid silver (e.g., silver nanoparticles) as the ultimate antimicrobial compound.
